# Peri-Implant Bone Loss and Peri-Implantitis: A Report of Three Cases and Review of the Literature

**DOI:** 10.1155/2016/2491714

**Published:** 2016-10-19

**Authors:** Vanchit John, Daniel Shin, Allison Marlow, Yusuke Hamada

**Affiliations:** Department of Periodontics and Allied Dental Program, Indiana University School of Dentistry, Indianapolis, IN 46202, USA

## Abstract

Dental implant supported restorations have been added substantially to the clinical treatment options presented to patients. However, complications with these treatment options also arise due to improper patient selection and inadequate treatment planning combined with poor follow-up care. The complications related to the presence of inflammation include perimucositis, peri-implant bone loss, and peri-implantitis. Prevalence rates of these complications have been reported to be as high as 56%. Treatment options that have been reported include nonsurgical therapy, the use of locally delivered and systemically delivered antibiotics, and surgical protocols aimed at regenerating the lost bone and soft tissue around the implants. The aim of this article is to report on three cases and review some of the treatment options used in their management.

## 1. Introduction

Implant supported restorative treatment has led to increased treatment options for patients who are either partially or completely edentulous. However, it has become evident that while this treatment is successful in many patients, implant supported restorations are not free of postplacement complications. Peri-implantitis is defined as an inflammatory process affecting the supporting hard and soft tissue around an implant in function, leading to loss of supporting bone. Peri-implant mucositis is defined as reversible inflammatory changes of the peri-implant soft tissues without any bone loss [1, 2]. The prevalence of peri-implant mucositis and peri-implantitis has ranged from 19 to 65% and 1 to 47%, respectively [2–4]. The most common etiological factors associated with the development of peri-implantitis are the presence of bacterial plaque and host response [5]. The risk factors associated with peri-implant bone loss include smoking combined with IL-1 genotype polymorphism, a history of periodontitis, poor compliance with treatment and oral hygiene practices, presence of systemic diseases affecting healing, cement left behind following cementation of the crowns, lack of keratinized gingiva, and previous history of implant failure [6].

The treatment of peri-implant disease must include decontamination of previously exposed or infected implant surfaces. However, current evidence has shown that nonsurgical therapy for peri-implantitis is minimally effective even with the adjunctive use of locally delivered or systemic antibiotics [5, 6]. Surgical access is usually required. The primary objective of surgical intervention is to allow the surgeon to instrument the implant surface and to perform debridement and decontamination. Decontamination and detoxification of the implant surface can be performed chemically or mechanically. Reported methods include the use of air-power abrasives, lasers, saline wash, ultrasonic use, and the use of chlorhexidine and hydrogen peroxide among others. These are usually combined with flap surgery [4, 7].

Several case report series have shown short- and long-term stability of soft tissue attachment along with radiographic bone fill following surgical procedures that have also included the use of bone regenerative procedures with barrier membranes, bone substitutes, and growth factors, such as enamel matrix derivative or platelet derived growth factors [8, 9]. However, there has been no clear definition in the existing literature on reosseointegration. Simonis et al. [10] conducted a systematic review seeking evidence of reosseointegration after treatment of peri-implantitis on contaminated implant surfaces. The authors concluded that reosseointegration is possible on a previously contaminated implant surface. These results were found in experimentally induced peri-implantitis defects following therapy. The amount of reosseointegration varied considerably within and between studies. Implant surface characteristics may influence the degree of reosseointegration. Surface decontamination alone cannot achieve substantial reosseointegration on a previously contaminated implant surface. No method predictably achieved the complete resolution of the peri-implant defect [11]. Esposito indicated in his Cochrane review that there is no reliable evidence suggesting the most effective intervention for treating peri-implantitis [12].

This article discusses two cases of peri-implant bone loss and one case of peri-implantitis. The differentiation in the terms is because in Cases 1 and 2 bone loss around the implant had occurred before the implants were restored. In Case 3, the patient presented with peri-implantitis many years after the implant was placed. The aim of the three case reports is to discuss the treatment of peri-implant bone loss and peri-implantitis to illustrate treatment modalities and to suggest a treatment protocol for surgical regenerative procedures for peri-implant bone loss.

## 2. Case Reports

**Figure 1:**
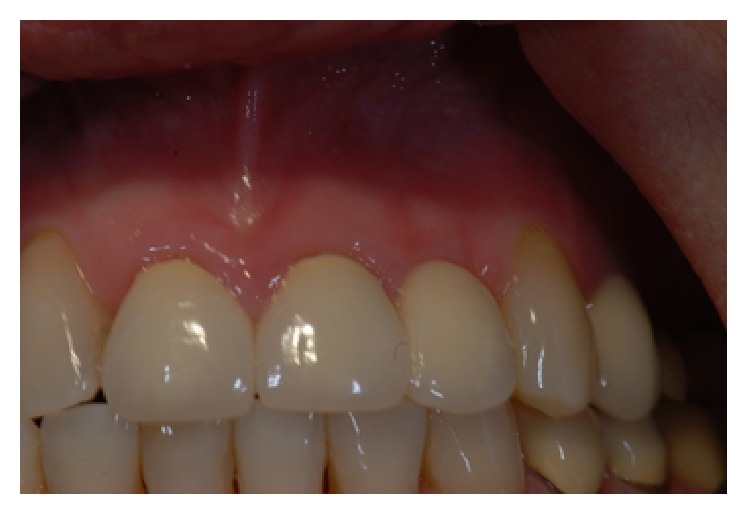
Initial presentation of the cantilever bridge in the maxillary anterior region.

**Figure 2:**
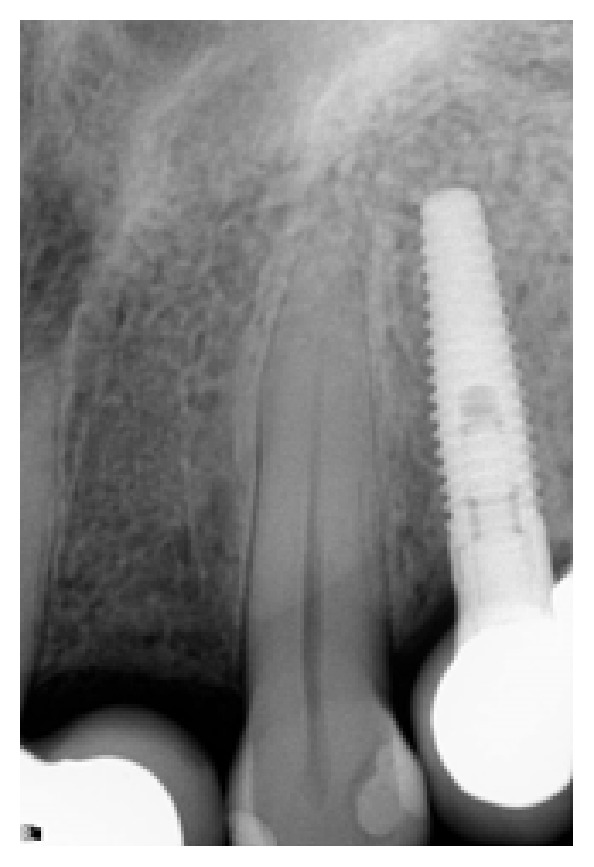
Periapical radiograph of the maxillary anterior region.

**Figure 3:**
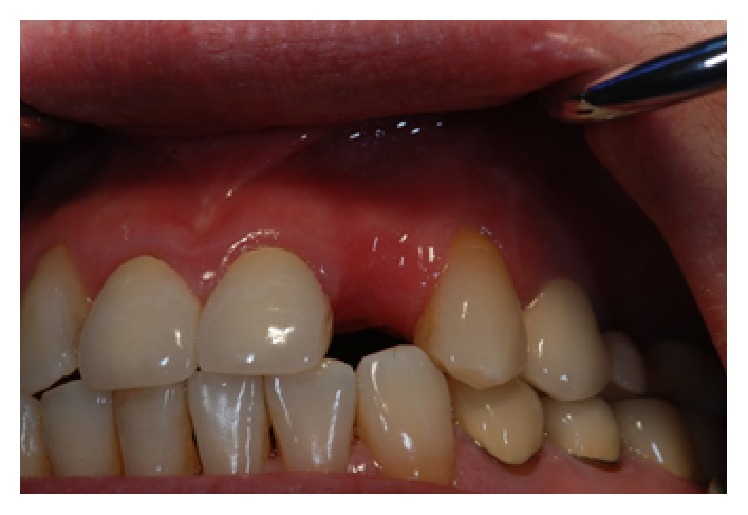
Following the sectioning of the cantilever bridge.

**Figure 4:**
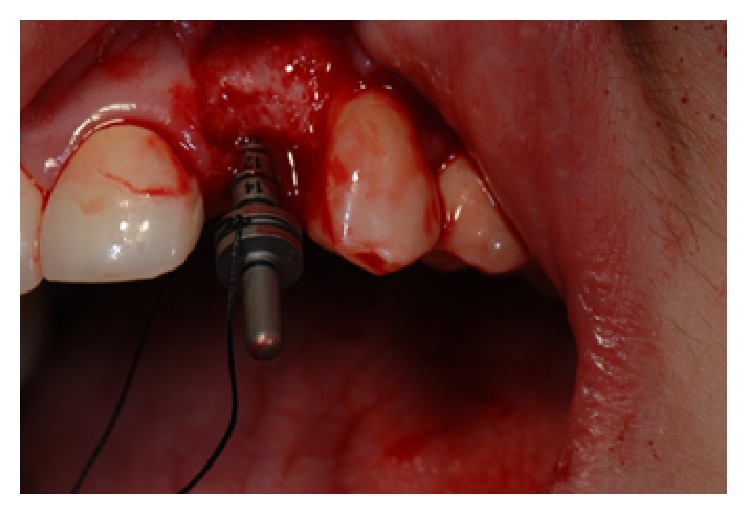
Placement of a paralleling pin to check angulation of the implant.

**Figure 5:**
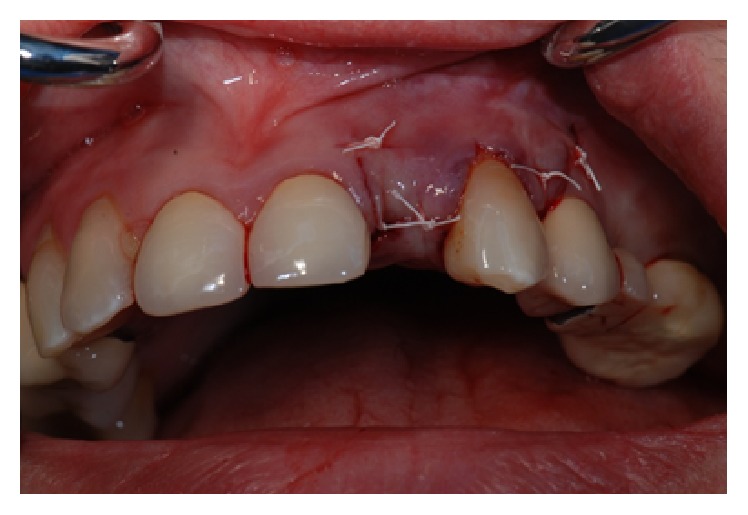
Following implant placement and suturing.

**Figure 6:**
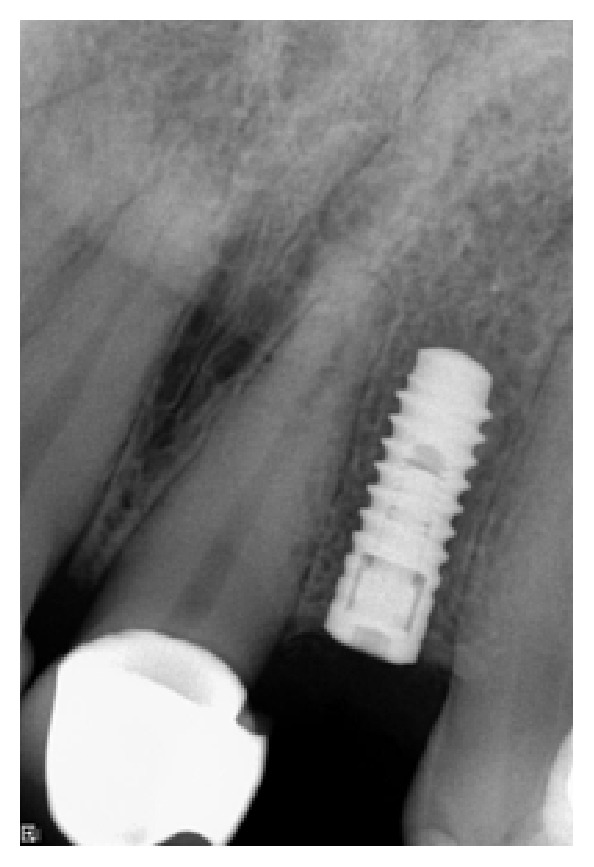
Periapical radiograph following implant placement.

**Figure 7:**
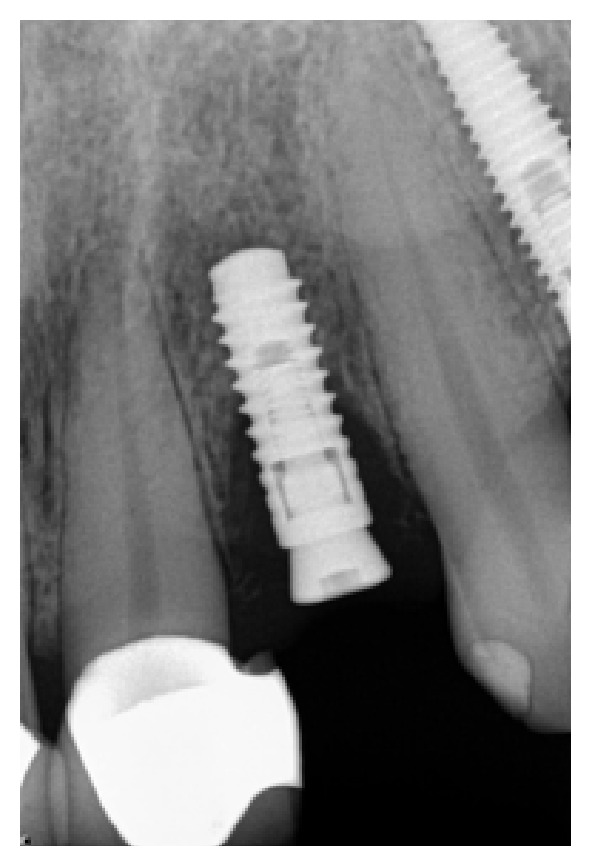
Following a healing phase of about 3 months, vertical bone loss around the implant was noted.

**Figure 8:**
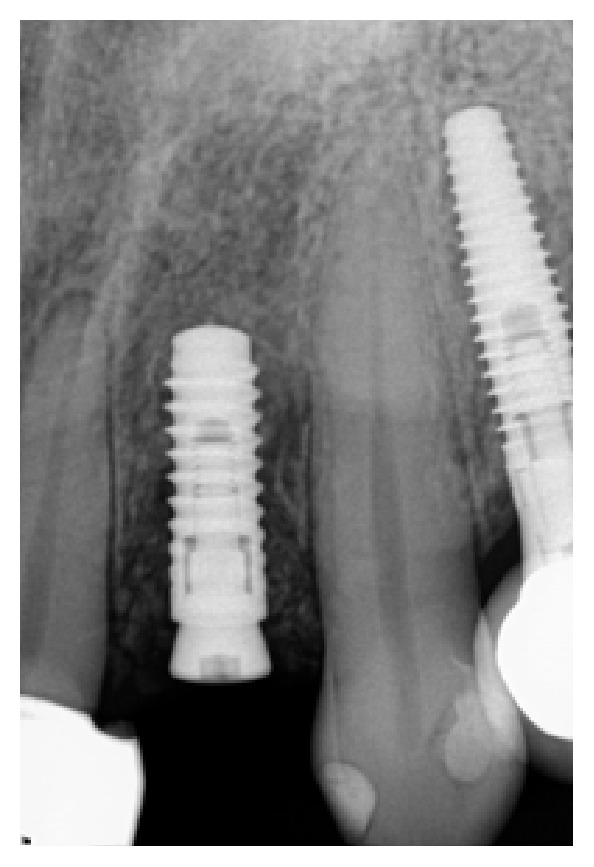
Following the treatment of implant site with EDTA, FDBA, and Emdogain®.

**Figure 9:**
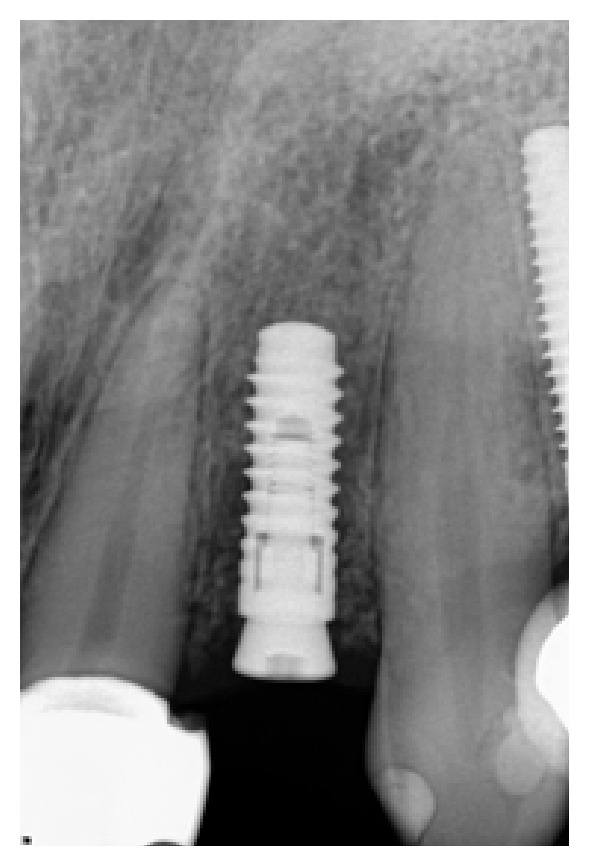
PA radiograph 4 months following bone grafting around the implant.

**Figure 10:**
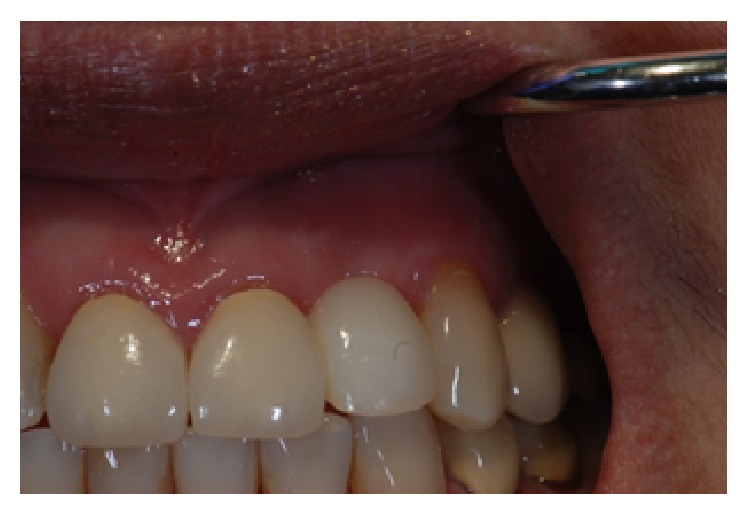
Provisional crown on the implant.

**Figure 11:**
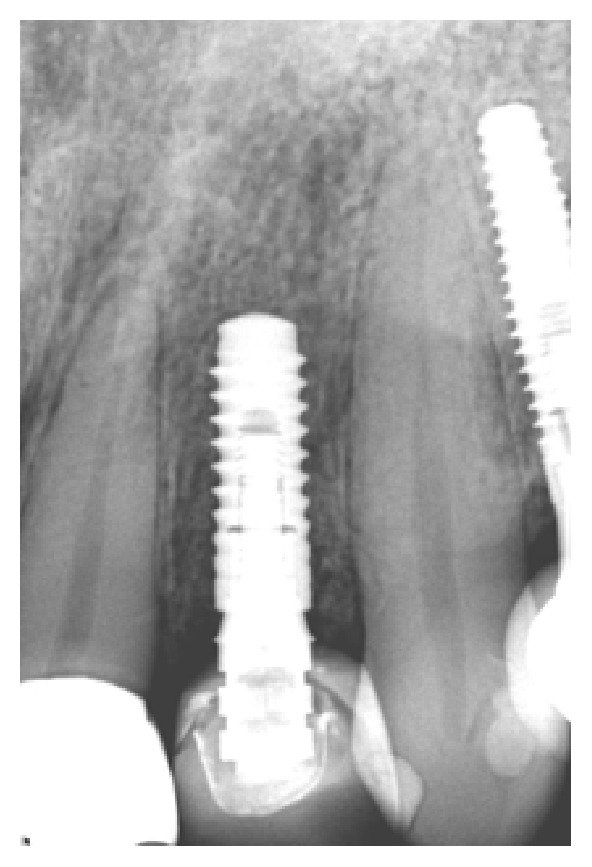
PA radiograph of provisional crown.

**Figure 12:**
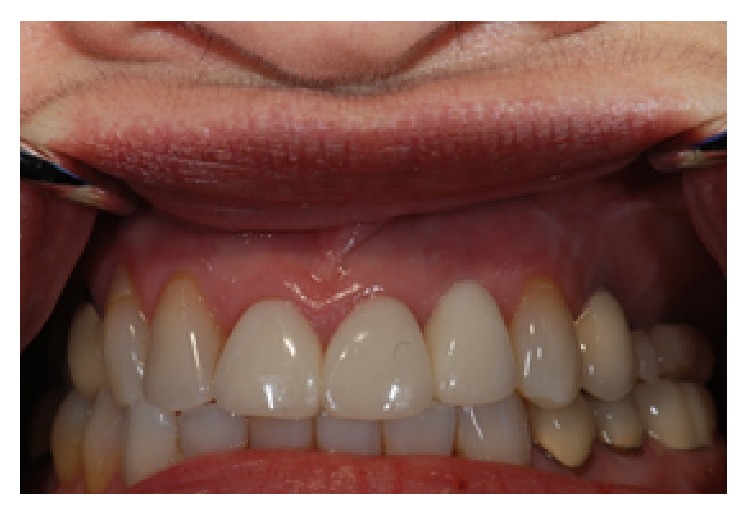
Final restoration of the implant in the #10 region.

**Figure 13:**
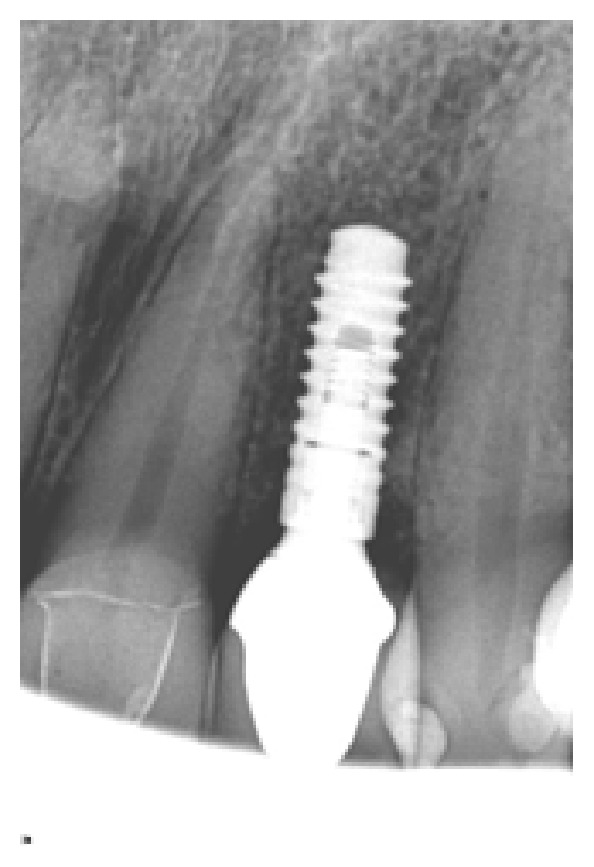
One year following implant restoration.

*Case 1 (Successful Treatment of Peri-Implant Bone Loss)*. A 48-year-old female, with no significant medical history, presented to the dental office as she was unhappy with her anterior cantilever bridge in the upper anterior region (Figures 1 and 2). Her initial appointment was for a consultation and periodontal exam. Her periodontal findings were normal with only isolated areas of mild gingival inflammation. Following the initial consultation, the patient was referred to a prosthodontist to section the bridge and to fabricate a provisional partial denture and a surgical template for implant placement (Figure 3). The implant was placed using a standard protocol (Figure 4). The implant used was a Straumann Roxolid® implant 3.3 mm in diameter and 10 mm in length. The area was sutured and the patient was given routine postoperative instructions. The patient was prescribed Amoxicillin 500 mg, 21 capsules to take 1 capsule three times a day for 7 days. Pain control included using Ibuprofen 600 mg every 6–8 hours for days 1 and 2 and then as needed after that. Healing appeared to proceed uneventfully (Figures 5 and 6). However, at second-stage surgery, which was done at 3 months following implant placement, vertical bone loss was noted on the mesial and distal aspects of the implant (Figure 7). It was decided to treat the site using the following protocol: the use of titanium curettes to instrument the implant surface and application of ethylenediaminetetraacetic acid (EDTA) twice for 2 minutes. The implant surface was then rinsed with normal saline following each application. The site was grafted with freeze-dried bone allograft (FDBA) combined with Emdogain (Figure 8). A similar post-op protocol was followed. Patient called the next day following surgery and indicated that she had fallen at home and hit her lip and that the site of surgery was bleeding. Following another visit, a second dose of antibiotics, Clindamycin 150 mg, 21 caps, 1 capsule three times a day for 7 days, was prescribed. Healing proceeded uneventfully. A periapical radiograph was taken at about 4 months (Figure 9). The patient was referred back to the prosthodontist for the fabrication of a provisional crown (Figures 10 and 11). Following a period of about 4 months, the patient had her final restoration fabricated (Figures 12 and 13). She is currently on a 6-month dental prophylaxis schedule.

**Figure 14:**
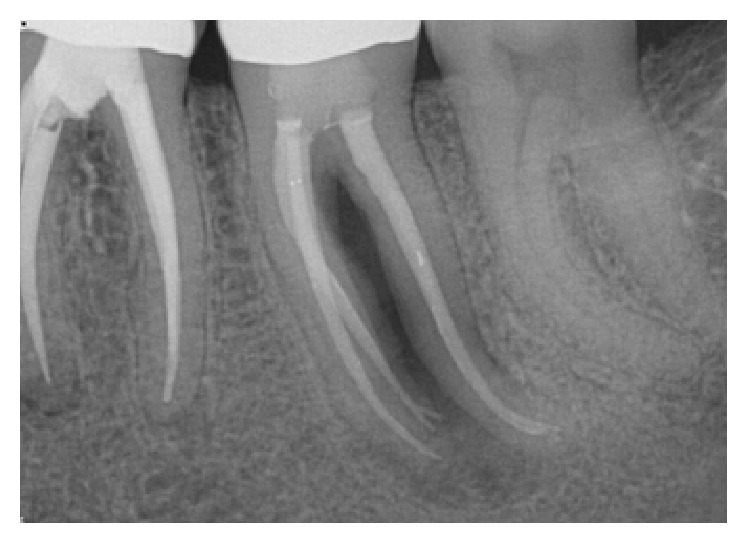
Tooth #18 presented with a hopeless prognosis.

**Figure 15:**
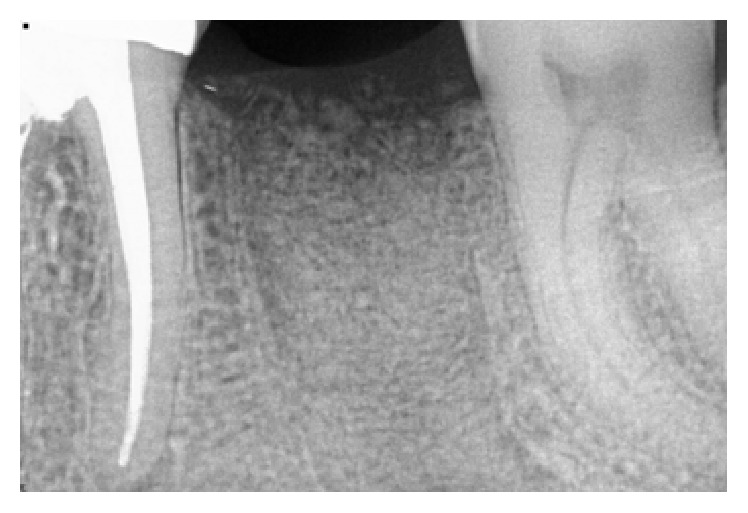
Following socket grafting—healing at 3 months.

**Figure 16:**
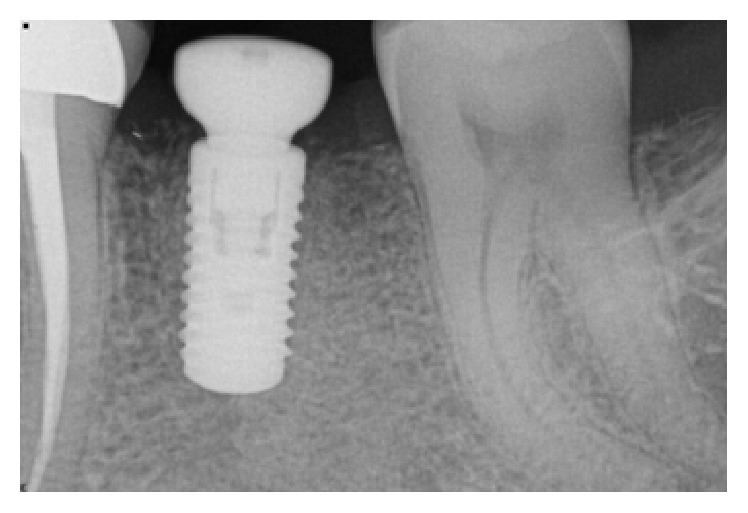
Implant was placed at 4 months using a flapless approach.

**Figure 17:**
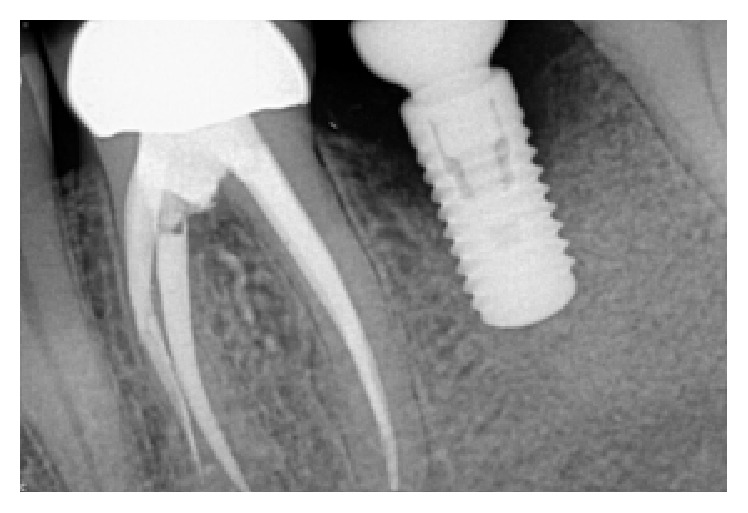
Presence of peri-implant bone loss.

**Figure 18:**
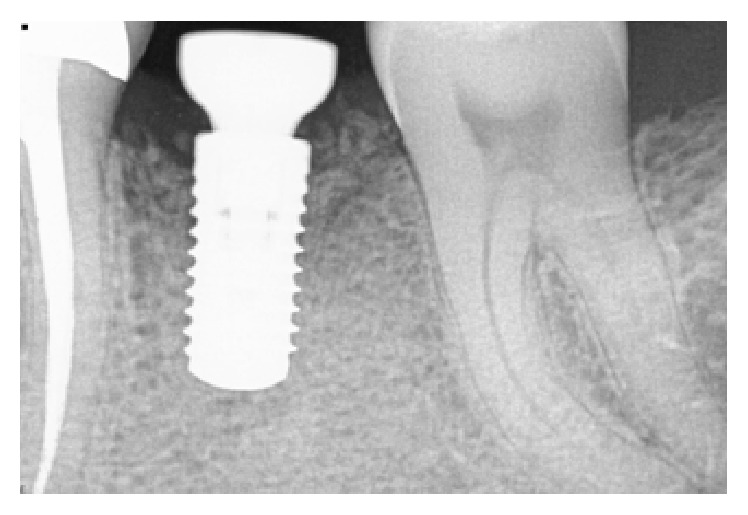
Six months following grafting of the site with freeze-dried bone allograft and a resorbable membrane.

**Figure 19:**
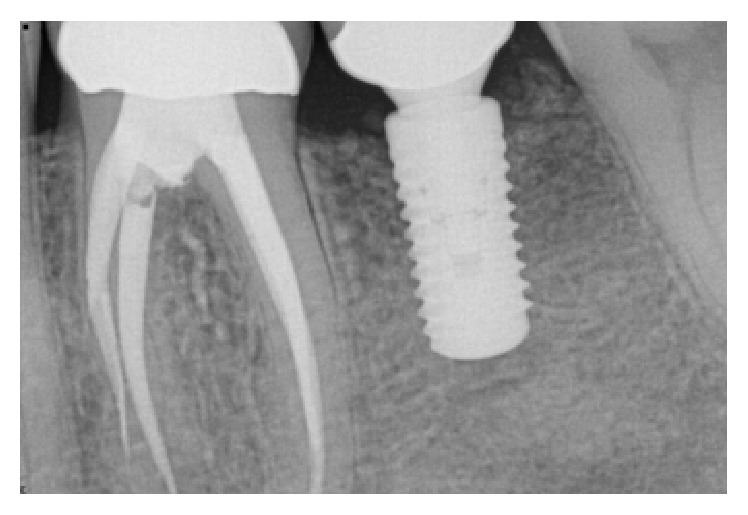
The implant was restored following a healing period of 6 months. The patient has been followed for 1 year now.

*Case 2 (Successful Treatment of Peri-Implant Bone Loss)*. A 58-year-old healthy male was referred to the dental clinic to evaluate tooth #18 (Figure 14) which presented with a cracked root and significant interradicular radiolucency. The tooth was deemed to have a “hopeless prognosis.” The plan was to extract the tooth and perform “socket grafting” to prepare the site for a future implant supported restoration. The tooth was sectioned and, following extraction, the socket was curetted and then grafted with freeze-dried bone allograft mixed with calcium sulfate. Calcium sulfate was also used as a barrier over the bone graft material (Figure 15). The patient was prescribed Amoxicillin 500 mg, 21 capsules to take 1 capsule three times a day for 7 days. Pain control included using Ibuprofen 600 mg every 6–8 hours for days 1 and 2 and then as needed after that. Following a healing period of four months, a flapless approach was used to place a 4.8 × 8 mm Straumann Roxolid implant in the site (Figure 16). Healing appeared to proceed uneventfully. A similar post-op protocol was followed. However, at second-stage surgery, bone loss was noted around the implant (Figure 17). Just as in Case 1, it was decided to treat the site using the following protocol: the use of titanium curettes to instrument the implant surface and the application of ethylenediaminetetraacetic acid (EDTA) twice for 2 minutes. The implant surface was then rinsed with normal saline following each application. The site was grafted with freeze-dried bone allograft (FDBA) combined with Emdogain (Figure 18). A similar post-op protocol to what had been done previously was followed. A follow-up radiograph was taken at about 6 months (Figure 19). The patient was then seen by his general dentist and the final crown was fabricated. Healing appeared to be progressing satisfactorily. The crown was fabricated 6 months following surgery to treat peri-implant bone loss. The patient has been followed for one year following the restoration of the implant with satisfactory bone levels being maintained. 

**Figure 20:**
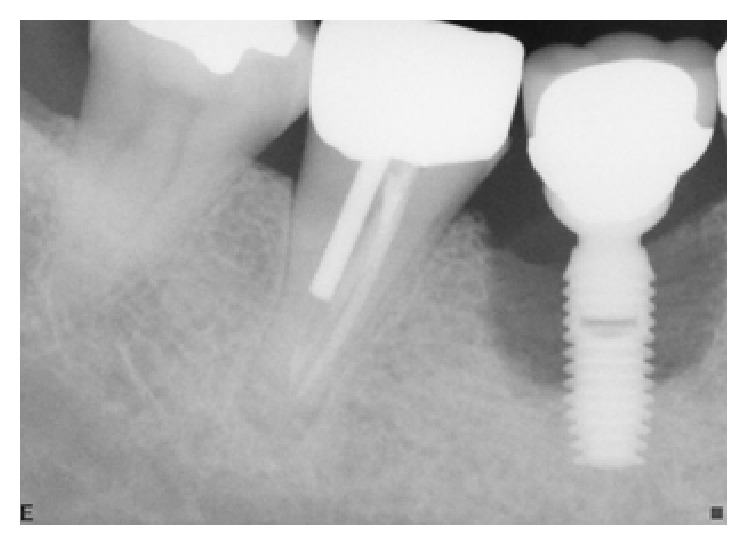
Peri-implant bone loss around the implant in the #30 site.

**Figure 21:**
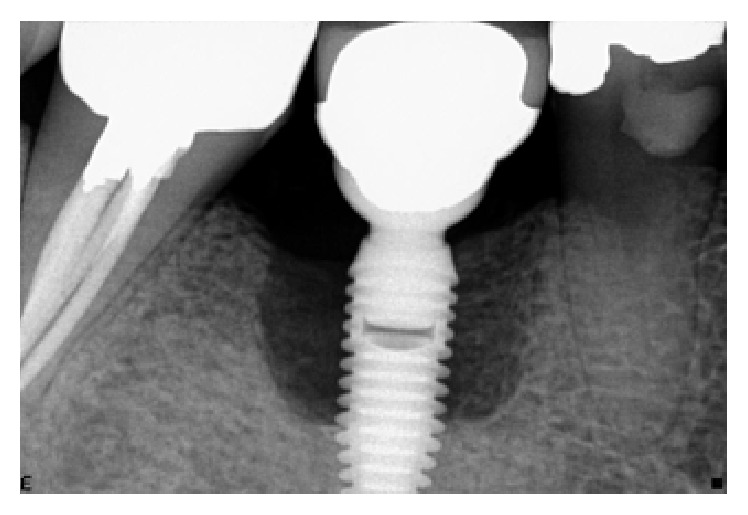
One year following treatment. Minimal to no improvement following surgical treatment.

**Figure 22:**
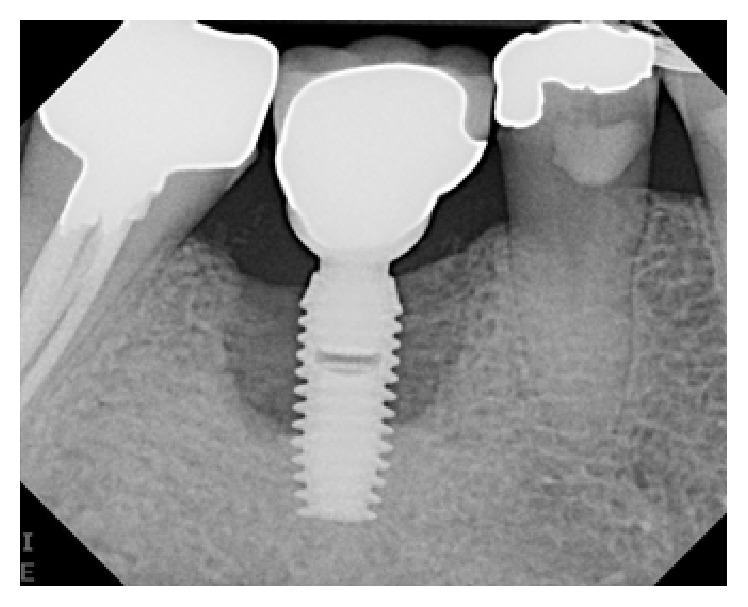
Four years following treatment. The site continued to present with inflammation and deep peri-implant probing depths.

**Figure 23:**
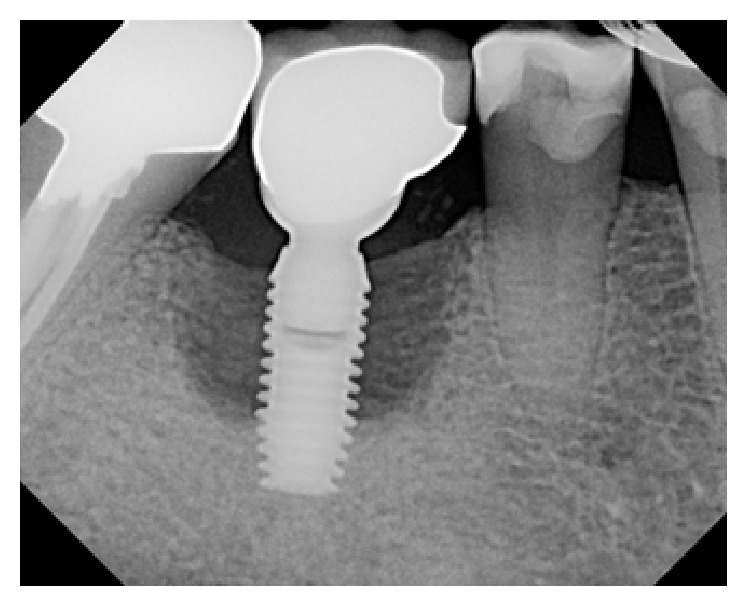
Six years following treatment. The patient has finally consented to having the implant removed and the site regrafted and evaluated following healing for possible future implant placement.

*Case 3 (Unsuccessful Treatment of Peri-Implantitis)*. A 50-year-old patient was referred to the dental office to evaluate the peri-implantitis around the implant supported restoration in the #30 region (Figure 20). The implant had been previously placed and restored in a different office in a different state. The patient was not sure how long ago treatment had been completed but reported that it had been at least 5 years since the implant was restored. Patient presented with deep peri-implant probing depths along with presence of exudate that could be expressed when the peri-implant tissues were palpated. The patient did not want to have the implant removed at that time. Initial treatment included a nonsurgical treatment phase that consisted of instrumentation around the implant with an ultrasonic instrument with an implant insert along with a titanium curette followed by subgingival irrigation with chlorhexidine gluconate (0.12%) and the placement of a locally delivered antimicrobial. Minocycline (Arestin®) was placed in the site to help with the healing process. However, the site continued to present with evidence of inflammation. Surgery to access the peri-implant defect was performed 6 months following nonsurgical treatment. The surgical treatment consisted of flap elevation, instrumentation with hand, and ultrasonic instruments, followed by the placement of freeze-dried bone allograft along with platelet-rich plasma. Following early healing, the level of inflammation had reduced. However about 1 year following treatment (Figure 21), there was evidence of increased inflammation along with the presence of some exudate in the site. Nonsurgical treatment was continued and the patient was compliant with keeping his appointments and maintaining his oral hygiene. At each appointment, the patient was counselled to have the implant removed. The patient was reluctant to have the implant removed. However, he finally consented to have the implant removed and the site prepared for a possible future implant supported restoration (Figures 22 and 23). Patient is scheduled to have the procedure done in 2017.

## 3. Discussion

The treatment of peri-implantitis and peri-implant bone loss presents a significant clinical problem facing many clinicians and their patients. The search for predictable treatment protocols is ongoing. Based on the two case reports presented and on the authors experience to date, the use of surface instrumentation of the exposed implants with an ultrasonic instrument using a special insert, combined with the application of EDTA for 2 minutes twice along with the use of Emdogain and freeze-dried bone allograft, has shown positive results.

The major challenge to effectively treating peri-implantitis fundamentally resides in our difficulty of conceptually tying together three equally important determinants that define the success or failure of an implant, that is, “implant survival rate,” “implant success rate,” and “implant complication rate.” Unfortunately, contemporary clinical implant reports tend to heavily stress on the “survival rate” and/or the “success rate” of an implant but very rarely draw attention to the “complication rate” that comes with defining the failure of an implant. This has both good and bad unintended consequences. On the positive side, since both “survival rate” and “success rate” have been shown to be exceptionally high, clinicians are inclined to revolve their entire treatment philosophy around the view that an endosseous implant is the gold standard when it comes to replacing an individual tooth. Yet, on the flip side of the coin, the overreliance of making a treatment decision on the basis of “survival rate” and/or “success rate”* alone* can negatively reinforce the misconceived assumption that dental implants are infallible. In other words, a dental implant will* never* fail. This notion is far from the truth! And, it becomes even more concerning when one considers that, with the growing number of implants being placed by dentists, there is also a concomitant rise in the number of implant-related complications. Thus, while both “survival rate” and “success rate” are important considerations, the overreliance of building our entire treatment philosophy on these two overarching factors means that we are putting too much weight on the success and/or survivability of the implant, but little on factoring the complications which could have clinically significant consequences on the long-term success and failure of the implant. The importance of the “complication rate” was highlighted in a 10–16-year clinical study which reported the incidence of implant biological complications, namely, peri-implantitis, to be approximately 17% [10]. A similar finding was reported in a recent meta-analysis which revealed a weighted mean prevalence of 22% for peri-implantitis [3]. To put these numbers in perspective, the American Academy of Implant Dentistry (AAID) has reported that approximately 5.5 million implants were placed in the United States in 2006 alone. Thus, using the numbers provided above, it can be inferred that over one million dental implants that were placed ten years ago are at risk of peri-implantitis at present day. This is quite a sobering statistic and, if accurate, is a harbinger of what is to come.

These three case reports are powerful and vivid reminders of the overwhelming destructive consequences of peri-implantitis. At the same time, these case reports illustrate that peri-implantitis—despite being difficult to treat—is still a treatable condition which demands early treatment and early intervention. For instance, Cases 1 and 2 clearly demonstrate that early detection and immediate treatment was critical in arresting peri-implant breakdown and in achieving an optimal regenerative outcome. On the other hand, Case 3 is a prime example of what could possibly happen if surgical intervention is delayed. Over time, the peri-implant infection festers and the prognosis of the implant worsens to the point that any future surgery is for naught. Hence, the common theme from all three cases is that treatment intervention will only have its maximum therapeutic benefits if it is detected early and promptly addressed with appropriate means. Only then can we see an outcome that reverses the effects of peri-implantitis, improves the survivability of the implant, and reduces the risk of complications that could potentially threaten the health of the implant.

Peri-implantitis is a complex multifactorial disease that shares many clinical characteristics and risk factors associated with periodontitis [13, 14]. As such, conventional treatment of peri-implantitis follows along the same lines of surgical treatment of periodontitis. In its most basic form, therapeutic intervention can be subdivided into two phases: (1) the anti-infective phase and (2) the regenerative phase. Similar to treating periodontitis, the primary objective of the anti-infective phase is mechanical decontamination of the implant surface while the primary objective of the regenerative phase is to establish an environment that is conducive to reosseointegration.

From a theoretical standpoint, the rationale for both the anti-infective phase and the regenerative phase has a strong basis. Yet, what remains open to discussion is determining which anti-infective strategy predictably achieves the best therapeutic outcome. Currently, there is no consensus as to which anti-infective strategy is the gold standard. Several conventional anti-infective modalities have been proposed. Mechanical scaling with plastic curettes or titanium curettes and ultrasonic scaling with an implant insert tip are examples of commonly used anti-infective strategies employed to treat peri-implantitis. As shown in the three case reports, the regenerative phase of treatment was preceded by some sort of mechanical debridement and/or ultrasonic debridement of the implant surface. The benefit of employing this type of anti-infective strategy is that it is gentler to the implant surface (less risk of damage to the surface). The limitation, however, is that even with thorough debridement there is no guarantee that the operator can entirely remove the plaque biofilm that has contaminated the implant surface. Furthermore, anti-infective therapy, when done thoroughly and meticulously, can be time-consuming and lead to both patient and operator fatigue. Another recently proposed anti-infective strategy is the use of titanium brushes (Straumann TiBrush™). The titanium brush has a standard dental coupling that fits onto a surgical hand piece while the other side has thin titanium bristles. When activated, the titanium bristles brush the implant surface in a clockwise and counterclockwise manner, thereby sweeping the plaque biofilm away from the implant. The titanium brush appears to be a more efficient means of removing the plaque biofilm when used in conjunction with manual implant scalers. In fact, in a recent preclinical study investigating the* in vivo* effects of the titanium brush on ligature-induced experimental peri-implantitis, it was found that the titanium brush resulted in a statistically significant reduction in inflammation and statistically significant improvement in bony defect fill [15]. While this may be a promising anti-infective strategy, additional studies are required to further support the use of titanium brushes. Other types of anti-infective strategies have been proposed, such as using an air-powder abrasive system with sodium bicarbonate and newer treatment modalities, such as laser therapy and photodynamic therapy. These anti-infective strategies may hold the key to providing results that are superior to conventional therapy. However, like conventional anti-infective strategies, they require further analyses to clarify their impact on halting the devastating consequences of peri-implantitis.

Similar to anti-infective therapy, there is a lack of a gold standard regenerative approach. Current peri-implant regenerative strategies follow along the same path as guided tissue regeneration [7, 10]. Yet, what makes peri-implant regenerative therapy a challenge is the multiple factors that can influence the success or failure of the outcome, namely, surface topography, severity of the peri-implantitis inflammatory lesion, and the therapeutic effectiveness of the type of anti-infective therapy implemented. Ongoing research is being conducted to determine which regenerative protocol offers the best chance in terms of facilitating reosseointegration. But even then, the predictability of a regenerative outcome is predicated on the effectiveness of anti-infective therapy. In all three case reports, the clinician performed exhaustive and thorough surgical debridement of the implant surface. Yet, in spite of the thoroughness of implant debridement, only 2 of the three cases resulted in a successful regenerative outcome. The most plausible explanation would be that anti-infective therapy in Case 3 was inadequate in eliminating the pathogenic plaque biofilm from the roughened implant surfaces. This would suggest that successful treatment of peri-implantitis depends more on the quality of surgical debridement, rather than the selection of a certain type of regenerative material and/or the regenerative technique.

## 4. Conclusions and Practical Implication

Cases 1 and 2 were examples of patients who presented with evidence of early peri-implant bone loss following implant placement. These patients responded well to the treatment procedures that were aimed at restoring the lost bone tissue. Case 3 was an example of a patient who presented with long-standing inflammation and bone loss. The difference between successful treatment and failure may revolve around the degree of chronicity associated with the bone loss. Early detection and treatment of mucositis, peri-implant bone loss, and peri-implantitis appear to be key factors that determine the prognosis of implant supported restorations. The authors conclude that careful patient selection and experienced clinicians involved with the surgical and restorative phases of treatment combined with regular clinical and radiographic examination around implant supported restorations are the key to long-term clinical and functional success for implant supported restorations.
